# Nrf2 signalling and autophagy are involved in diabetes mellitus-induced defects in the development of mouse placenta

**DOI:** 10.1098/rsob.160064

**Published:** 2016-07-06

**Authors:** Mei-yao He, Guang Wang, Sha-sha Han, Ya Jin, He Li, Xia Wu, Zheng-lai Ma, Xin cheng, Xiuwen Tang, Xuesong Yang, Guo-sheng Liu

**Affiliations:** 1 Department of Pediatrics and Neonatology, Institute of Fetal-Preterm Labor Medicine, The First Affiliated Hospital, Jinan University, Guangzhou 510632, People's Republic of China; 2 Division of Histology and Embryology, Key Laboratory for Regenerative Medicine of the Ministry of Education, Medical College, Jinan University, Guangzhou 510632, People's Republic of China; 3 Postdoctoral Research Station of Chinese and Western Integrative Medicine, Institute of Integrated Traditional Chinese and Western, Medical College, Jinan University, Guangzhou 510630, People's Republic of China; 4 Department of Biochemistry and Genetics, School of Medicine, Zhejiang University, Hangzhou 310058, People's Republic of China

**Keywords:** pregestational diabetes mellitus, placenta, trophoblast cell, ROS, Nrf2, autophagy

## Abstract

It is widely accepted that diabetes mellitus impairs placental development, but the mechanism by which the disease operates to impair development remains controversial. In this study, we demonstrated that pregestational diabetes mellitus (PGDM)-induced defects in placental development in mice are mainly characterized by the changes of morphological structure of placenta. The alteration of differentiation-related gene expressions in trophoblast cells rather than cell proliferation/apoptosis is responsible for the phenotypes found in mouse placenta. Meanwhile, excess reactive oxygen species (ROS) production and activated nuclear factor erythroid2-related factor 2 (Nrf2) signalling were observed in the placenta of mice suffering from PGDM. Using BeWo cells, we also demonstrated that excess ROS was produced and Nrf2 signalling molecules were activated in settings characterized by a high concentration of glucose. More interestingly, differentiation-related gene expressions in trophoblast cells were altered when endogenous Nrf2 expression is manipulated by transfecting Nrf2-wt or Nrf2-shRNA. In addition, PGDM interferes with autophagy in both mouse placenta and BeWo cells, implying that autophagy is also involved, directly or indirectly, in PGDM-induced placental phenotypes. Therefore, we revealed that dysfunctional oxidative stress-activated Nrf2 signalling and autophagy are probably responsible for PGDM-induced defects in the placental development of mice. The mechanism was through the interference with differentiation-related gene expression in trophoblast cells.

## 1. Introduction

As shown in the literature, pregnant women with either pre-existing diabetes mellitus or gestational diabetes mellitus (GDM) are at higher risk of carrying a fetus with congenital anomalies including phocomelia, cardiac malformations, macrosomia and central nervous system malformations [1,2]. Among the diabetes mellitus-induced congenital malformations, congenital heart disease and anomalies of the nervous system are predominant, posing 3.4 times higher risk and 2.7 times higher risk, respectively [3]. Note that this may be because both the cardiovascular and nervous systems are formed in the early developmental stage, making them more vulnerable to external harmful factors. These congenital development defects comprise pulmonary atresia, double outlet right ventricle, tetralogy of Fallot and fetal cardiomyopathy [4]. However, the exact aetiology and pathogenesis for diabetes mellitus-induced congenital development defects remains controversial.

Hyperglycaemia, which promotes the generation of excess reactive oxygen species (ROS), is proposed to be the most important teratogen leading to congenital disease formation [5]. The well-substantiated excess production of ROS may be partially responsible for the development of congenital defects [6]. Moreover, the congenital defects of fetal organogenesis were caused by either direct damage or indirect impairment arising from hyperglycaemia, both of which affect placental development. Hyperglycaemia will lead to placental dysfunction and subsequent pregnancy complications [7], and GDM has been associated with alterations in placental anatomy and physiology. These alterations are mainly based on changes on the micro-anatomical and/or even molecular level [8].

In mammals, the regulation of autophagy by amino acids, and also by the hormone insulin, has been extensively investigated, but knowledge about the effects of glucose is more limited. Under certain conditions, autophagy can be activated by glucose [9]. Autophagy is critical to the process of development in mouse models [10], it is involved in development of the human placenta, and changes in oxygen and glucose levels participate in regulation of autophagic changes in cytotrophoblast cells [11]. Furthermore, autophagy plays important roles during starvation in the mouse fetus when the supply of amino acids through the placenta is suddenly cut off [12]. Therefore, in this study we focus on the impact of hyperglycaemia on placental development.

Placenta is the physiological and mechanical connection between fetal and maternal tissues for the respiration, nutrition and excretion of the fetus. The placenta also functions as an immunological barrier, fighting off internal infection. It transports pathogens/drugs and stores some fats, glycogen and iron. Finally, the placenta executes critical endocrine functions, producing hormones throughout pregnancy [13].

Mouse placenta is often used for placental study as it shares many similarities with human placenta, although certain unconformities in genetic regulation exist between them [14]. Histologically, the placenta is composed of maternal and fetal portions. The fetal portion of the placenta is derived from extraembryonic mesoderm (allantois). At E8.5, the allantois and the chorion join together in a process called chorioallantoic attachment. Soon afterwards, the chorion starts folding to form the villi, and forming a space into which the fetal blood vessels grow from the allantois [15]. The maternal portion of placenta derives from the maternal vasculature and the decidual cells of the uterus. The two portions are closely allied, functioning as a fetomaternal blend as villi are anchored to the decidua basalis to fulfil placental physiological functions. During placentation, the trophoblast lineage provides most of the structural components for the placenta and is indispensable to contact between fetal and maternal blood [14]. Therefore, a series of regulatory genes of the trophoblast lineage are seriously attended throughout the different developmental stages of development [16].

In this study, we demonstrate that hyperglycaemia could dramatically affect the development of mouse placenta. Furthermore, we demonstrated that the phenotypes in mouse placenta are closely related to the imbalance of oxidative stress-activated nuclear factor-like 2 (Nrf2) signalling and autophagy.

## 2. Material and methods

### 2.1. Experimental animals

The Kunming mice used in this study were obtained from the Laboratory Animal Centre of Sun Yat-Sen University (Guangzhou, China). Eight-week-old female mice were used to induce diabetes mellitus by injecting STZ (Sigma, MO, USA) dissolved in 0.01 M citrate buffer at a pH of 4.5 and a dose of 75 mg kg^−1^ body weight for three consecutive days. Blood glucose levels were measured 7 days after STZ injection by Roche Accu-Chek Aviva Blood Glucose System (Roche, USA). Diabetes mellitus was defined as a non-fasting blood glucose level greater than 288 mg dl^−1^ (16 mM) [17]. Control mice were maintained euglycaemic prior to and during pregnancy (4–8 mM). Two female mice were housed with one normal male mouse overnight in a cage. The day when vaginal plugs were observed was designated as embryonic day 0.5 (E0.5). During pregnancy, blood glucose levels were monitored every 6 days. At E13.5 and E18.5, the embryos were dissected by Caesarean section after the pregnant mice were anaesthetized by an intraperitoneal injecting pentobarbital (150 mg kg^−1^ body weight) [18]. The experiments were performed in triplicates for the three embryonic days, with 24 mice assigned to control and pregestational diabetes mellitus (PGDM) groups.

### 2.2. Placental morphological analysis

We examined whether PGDM altered the morphology of mouse placenta. The placentas were harvested at each assigned time. Placentas were photographed and then fixed in 4% paraformaldehyde, then dehydrated, embedded in paraffin wax and serially sectioned at 4 µm. For histology, the sections were de-waxed in xylene, rehydrated and stained with haematoxylin & eosin dyes (H&E) or periodic acid-Schiff dyes (PAS). The sections were photographed using a fluorescence microscope (Olympus IX50) linked to NIS-Elements F3.2 software. The average size (area) of the placental labyrinth and junctional zones were determined and evaluated through dividing the areas of labyrinth zone by the total area of placental transverse section, as previously described [19], a minimum of randomly five images from five samples were respectively assayed per group and per assigned time. The spongiotrophoblast layer was positive for PAS, which suggests that many of these spongiotrophoblasts were glycogen cells. The ratio of glycogen-positive area to total area was determined as previously described [20]. A minimum of randomly three images from five samples were respectively assayed per group and per assigned time.

### 2.3. Immunostaining

Immunostaining was performed on paraffin transverse sections against proliferating cell nuclear antigen (PCNA), light chain 3 (LC3B) and Nrf2 antibodies. Briefly, placental transverse sections were de-waxed in xylene, rehydrated and then heated in a microwave for antigen retrieval before exposure to the primary antibody with citrate buffer (pH = 6.0). Then, sections were immersed in 3% hydrogen peroxide for 10 min to block endogenous peroxidase. Non-specific immunoreactions were blocked using 5% inactivated goat serum in PBS for 30 min at room temperature. The sections were washed in PBS and incubated with PCNA (1 : 500, Santa Cruz, sc-7907, CA, USA), LC3B (1 : 200, Cell Signaling Technology, D11, MA, USA) and Nrf2 (1 : 200, Santa Cruz, sc722) antibodies overnight with shaking at 4°C. Following extensive washing, the sections were incubated in horseradish peroxidase (HRP) goat anti-rabbit IgG secondary antibody (1 : 400, EarthOx, 7074S, Millbrae, USA) for 3 h at room temperature in a dark box, and conjugated to DAB (Maixin, Fuzhou, China). After immunostaining, the sections were counterstained with haematoxylin. For intensity, analysis of PCNA expression was selected for semiquantitative analysis by H-SCORE [21].

### 2.4. TUNEL analyses

The extent of apoptosis in the placenta was established using an In Situ Cell Death Detection Kit (Roche, USA). Terminal deoxynucleotidyl transferase dUTP nick end labelling (TUNEL) staining, including the negative control, was performed according to instructions provided by the manufacturer, which we adapted for tissue labelling on the glass slides. The presence of TUNEL^+^ cells was established using image analysis software (Olympus, Japan). For intensity analysis of TUNEL expression was selected for semiquantitative analysis by *H-SCORE* [21], both control and experimental groups (*n* = 8 placentas for each group).

### 2.5. Cell culture and gene transfection

BeWo, the trophoblast-derived choriocarcinoma cell line, was attained from ATCC (American Type Culture Collection, CCL-98, USA). The cells were cultured in a humidified incubator with 5% CO_2_ at 37°C in six-well plates (1 × 10^6^ cells ml^−1^) containing HAM'S/F-12 (Myclone, USA) supplemented with 10% fetal bovine serum (Gibco, Gaithersburg, MD, USA), and exposed to 7 mM, 17 mM, 30 mM d-glucose (Sigma); 7 mM d-glucose was used as a control, and 30 mM mannitol acting as an osmolarity control. The cells were photographed using an inverted fluorescence microscope (Nikon, Ti-u, Japan) linked to NIS-Elements F3.2 software. After 72 h incubation, the immunofluorescent staining against Nrf2 (1 : 200, Santa Cruz, sc722), F-actin (1 : 500, Life Technologies, A12379, USA) and LC3B (1 : 200, Cell Signaling Technology, D11) was performed in the incubated BeWo cells. A minimum of five images were assayed per treatment group. For gene transfection, the BeWo cells were transfected by GFP, Nrf2-wt or Nrf2-shRNA with the help of lipofectamin 2000 (Invitrogen, CA, USA). Cells were plated to 50–70% confluence at the time of transfection and the preparation of plasmid DNA–lipid complexes, which were subsequently added to the cells. Additionally, the HG&Nrf2-shRNA group would be treated with high glucose before transfection. The insertion sequence for the Nrf2 shRNA is GGGCAAGATATAGACCTTGGTCAAGAGCCAAGGTCTATATCTTGCCTTTTTTGA and GCAGTCTTCATTTCTGCTAATCAAGAGTTAGCAGAAATGAAGACTGTTTTTTGA.

### 2.6. Cell counting kit-8 assay

Cell viability was assessed through a modified cell counting kit-8 (CCK8; Dojindo Molecular Technologies, Japan) assay. Briefly, 10 µl of CCK8 (5 g l^−1^) was added into 96-well plates and incubated continually for 4 h at 37°C. The absorbance values were measured at 450 nm using a BIO-RAD Model 450 Microplate Reader (BIO-RAD, CA, USA). Cell viability was indirectly established by the ratio of the absorbance value of 17 mM, 30 mM d-glucose or 30 mM mannitol-treated cells relative to the control (7 mM d-glucose). The final results were determined from analysing six independent experiments.

### 2.7. Measurement of intracellular reactive oxygen species

Intracellular ROS was determined using a non-fluorescent dye 2′7′-dichlorodihydrofluorescein diacetate (Sigma-Aldrich), which is oxidized by ROS generated by cells into a fluorescent dye 2′,7′-dichlorofluorescin. The control and high glucose-treated BeWo were incubated in the presence of 10 µm 2′7′-dichlorodihydrofluorescein diacetate for 20 min. The fluorescence was measured using a BD FACSAria (Becton, Dickinson and Company, Franklin Lakes, NJ, USA).

### 2.8. RNA isolation and RT-PCR

Total RNA was isolated from placental tissues or BeWo cells using EZNA. Total RNA Kit (OMEGA, Georgia, USA) according to the manufacturer's instructions. Reverse transcription to synthesize cDNA was accomplished using PrimeScript RT Reagent Kit with gDNA Eraser (Takara, Shiga, Japan). PCR amplification of the cDNA was performed using specific mouse primers shown in electronic supplementary material, figure S1. PCR was performed in a BIO-RAD S1000 Thermal cycler (BIO-RAD). The cDNAs were amplified for 40 cycles. One round of amplification was performed at 95°C for 5 s, at 56°C for 30 s and at 72°C for 30 s (TaKaRa, Japan). The PCR products (20 µl) were resolved on 2% agarose gels (Biowest, Spain) in a 1× TAE buffer (0.04 M Trisacetate and 0.001 M EDTA) and with GeneGreen Nucleic Acid Dye (TIANGEN, China). The reaction products were visualized using a transilluminator (SYNGENE, UK) and a computer-assisted gel documentation system (SYNGENE). The sets of primers used for RT-PCR are provided in the electronic supplementary material, figure S1. The ratio between the intensity of the fluorescently stained bands corresponding to the genes and β-actin was calculated to quantify the level of the transcripts for those genes mRNAs [15,22]. The RT-PCR result was representative of three independent experiments.

### 2.9. Western blotting

Western blotting was performed in accordance with a standard procedure using a polyclonal antibody that specifically recognized P53, PCNA, Kelch-like ECH-associated protein 1 (Keap1), Nrf2, NAD(P)H Dehydrogenase Quinone 1 (NQO1), Beclin1 and LC3B. The collected placental tissues or BeWo cells were frozen in the liquid nitrogen and kept at −80°C. Protein from the placental tissues or BeWo cells was isolated from tissue homogenates or cell lysates using a radio-immuno-precipitation assay (RIPA, Sigma) buffer supplemented with protease and phosphatase inhibitors. Protein concentrations were quantified with the BCA assay. The extracted protein was separated by 10% SDS-PAGE, and transferred onto a polyvinylidene difluoride (PVDF) membrane (Millipore, MA, USA). The membrane was blocked with 5% non-fat milk and then incubated with anti-P53 antibody (1 : 1000, Millipore, 2273812), PCNA antibody (1 : 1000, Santa Cruz, sc-7907), Keap1 antibody (1 : 1000, Santa Cruz, sc-33569), Nrf2 antibody (1 : 1000, Santa Cruz, sc722), NQO1 antibody (1 : 1000, Santa Cruz, sc-25591), Beclin1 (1 : 1000, Cell Signaling Technology, 3495), LC3B (1 : 1000, Cell Signaling Technology, D11), ATG7 antibody (1 : 500, BOSTER, BA3527-2, Wuhan, China), ATG5 antibody (1 : 300, BOSTER, BA3525-2), SQSTM1 (P62) antibody (1:500, BOSTER, BA2849) in TBS buffer at 4°C overnight. The loading control was β-actin antibody (1 : 3000, Proteintech, 60 008-1-1 g, Rosemont, USA). After incubation with the secondary antibody, either HRP goat anti-rabbit IgG (1 : 3000, EarthOx, 7074S) or HRP goat anti-mouse IgG (1 : 3000, EarthOx, 7076S), the blots were developed with SuperSignal West Femto Chemiluminescent Substrate (ThermoFisher, Rockford, USA), Gel Doc XR+ System (BIO-RAD). Quantity One (BIO-RAD) software was used to capture the chemiluminescent signals and analyse the data.

### 2.10. Data analysis

Data analyses and construction of statistical charts were performed using a GraphPad Prism 5 software package (Graphpad Software, CA, USA). The results are presented as the mean value (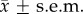). All data were analysed using ANOVA or *t*-test, which was employed to establish whether there was any difference between the control and experimental data. *p* < 0.05 was considered to be significantly different. The datasets supporting this article have been uploaded as part of the electronic supplementary material.

## 3. Results

### 3.1. Developmental defects in mouse placenta occurred in the presence of high levels of maternal glucose

**Figure 1.**
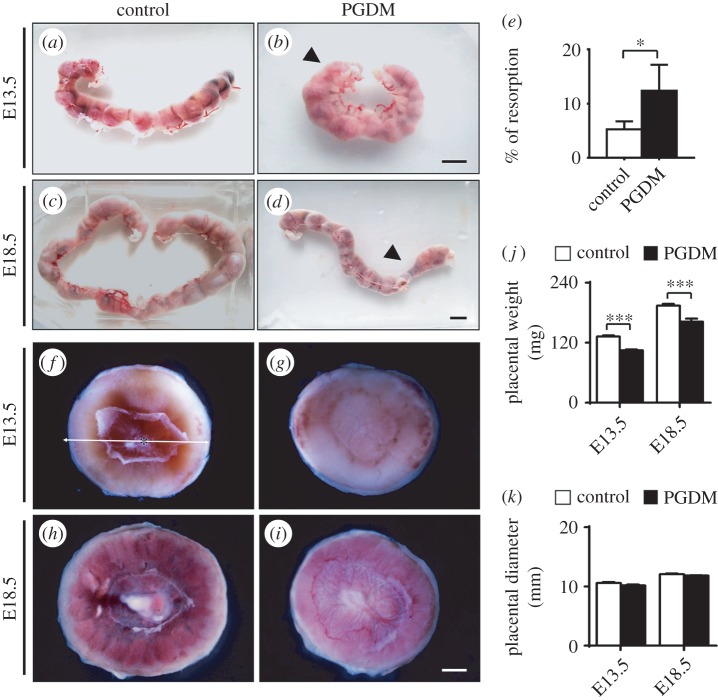
The mouse embryo numbers and morphological alteration of placenta in the presence of high levels of glucose. (*a*–*d*) The representative brood offspring from mice in control (*a*,*c*) and PGDM (*b*,*d*) groups at E13.5 (*a*,*b*) and E18.5 (*c*,*d*) embryonic days. (*e*) The bar chart compares the abortion rate of the controls (*n* = 34) and the PGDM group mice (*n* = 18). Resorption is defined as pregnancy loss or abortion. In other words, it stands for fetal deaths. (*f*–*i*) The representative appearance of placenta from control (*f*,*h*) and PGDM (*g*,*i*) group mice at E13.5 (*f*,*g*) and E18.5 (*h*,*i*) embryonic days. (*j*) The bar chart compares placental weights of control (*n* = 46) and PDGM (*n* = 46) group mice. (*k*) The bar chart compares placental diameters of control and PDGM group mice. PGDM, previous gestational diabetes. Scale bars = 10 mm in (*a*–*d*) and 2 mm in (*f*,*g*).

In our examination of the pregnant mice, we found that resorption or intermediate fetal death (indicated by arrows in figure 1*b*,*d*) happened in the presence of high glucose to a greater extent compared with these occurrences among the control groups; this difference was especially obvious during the late gestational stage (control (*n* = 34): 0.06 ± 0.02; PDGM (*n* = 18): 0.15 ± 0.06, *p* < 0.05; figure 1*a*–*e*). There are many causes of late termination in pregnancy. The defects in placental development are certainly among the more predominant causes. Here, the weight of placenta in the PGDM group significantly decreased compared with those in the control group of mice at both gestational ages E13.5 (control: 132.30 ± 2.34 mg, PGDM: 104.50 ± 2.08 mg, *p* < 0.001, *n* = 46 for each group) and E18.5 (control: 194.20 ± 3.754 mg, PGDM: 162.00 ± 6.20 mg, *p* < 0.001, *n* = 21 for each group; figure 1*f*–*j*), while there was no significant difference between control and PGDM placenta in terms of placental diameters (E13.5—control: 0.60 ± 0.15 mm, PGDM: 10.17 ± 0.15 mm, *p* > 0.05; E18.5—control: 12.10 ± 0.11 mm, PGDM: 11.86 ± 0.07 mm, *p* > 0.05, *n* = 13 for each group; figure 1*k*).

**Figure 2.**
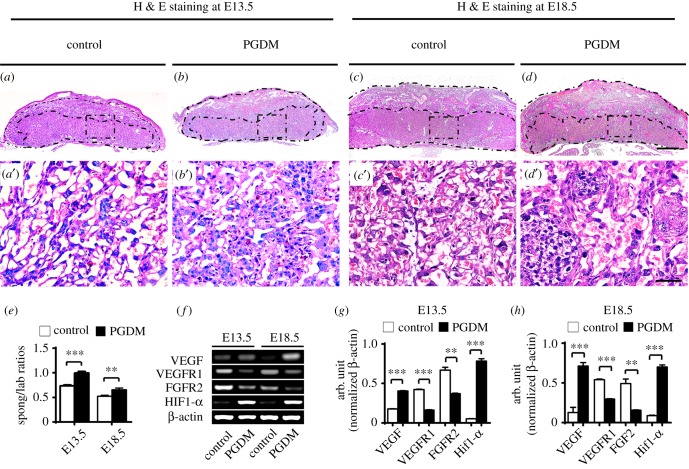
The alteration of the embryonic placental labyrinth and spongiotrophoblast thickness in the presence of high levels of glucose. (*a*–*d*) The representative H&E stained transverse sections from the control (*a*,*c*) and PGDM (*b*,*d*) group placenta at E13.5 (*a*,*b*) and E18.5 (*c*,*d*) embryonic days. (*a*′–*d*′) The high-magnification images were taken from the sites indicated by dotted squares in (*a*–*d*). (*e*) The bar chart compares the ratios of the placental spongiotrophoblast to labyrinth areas of the control and PDGM group placenta at E13.5 and E18.5 embryonic days (Spong/Lab). (*f*) The RT-PCR data shows the expressions of VEGF, VEGFR1, FGFR2 and HIf1-α in E13.5 or E18.5 placental tissues from the control and PDGM groups. (*g*,*h*) The bar charts show the ratios of the band arbitrary unit at the corresponding gene expression to the normalized β-actin from RT-PCR data in E13.5 (*g*) or E18.5 (*h*) placental tissues. Scale bars = 0.5 mm in (*a*–*d*) and 0.05 mm in (*a*′–*d*′).

According to the characteristics of the morphological structure of mice, the placenta could be histologically divided into three layers under transverse section as shown in figure 2*a*–*d*. In the representative H&E stained transverse sections of placenta, we could see that the ratio of placental junctional zone and labyrinth zone changed at both gestational ages E13.5 (control: 0.74 ± 0.02, PGDM: 0.99 ± 0.03, *p* < 0.001, *n* = 25 for each group) and E18.5 (control: 0.52 ± 0.02, PGDM: 0.66 ± 0.04, *p* < 0.01, *n* = 25 for each group) in the mice (figure 2*a*–*e*). It is suggested that in PGDM placenta, the relative area of the labyrinth zone was reduced while the relative area of the junctional zone was enhanced compared with the control group.

In addition, the mRNA expressions of VEGF, VEGFR1, FGFR2 and Hypoxia-inducible transcription factors (HIF1-α) in the placental tissues were assessed using RT-PCR. This assessment showed that VEGF and HIF1-α mRNA expressions were upregulated in PDGM placenta at both gestational age E13.5 (VEGF, *p* < 0.001; VEGFR1, *p* < 0.001; FGFR2, *p* < 0.01; HIF-1α, *p* < 0.001, *n* = 3 for each group) and gestational age E18.5 (VEGF, *p* < 0.001; VEGFR1, *p* < 0.001; FGFR2, *p* < 0.01; HIF-1α, *p* < 0.001, *n* = 3 for each group) compared with the control group (figure 2*f*–*h*). This suggests that the gene expression for angiogenesis in PDGM placenta was interfered with in comparison with the control group.

### 3.2. Cell proliferation and apoptosis in mouse placenta were altered by exposure to high maternal glucose levels

**Figure 3.**
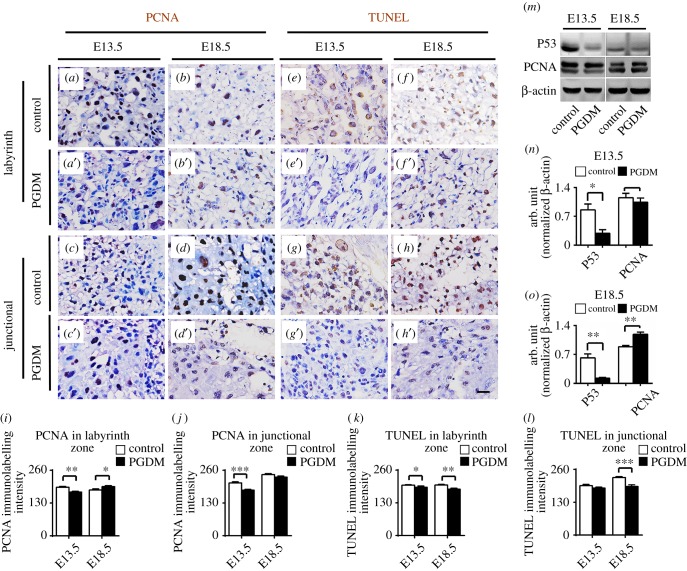
The assessment of cell proliferation and apoptosis of placental tissues in the presence of high levels of glucose. (*a*–*d*) The representative PCNA immunostained transverse sections of the labyrinthine (*a*,*b*) or junctional layers (*c*,*d*) from control placenta at E13.5 (*a*,*c*) or E18.5 (*b*,*d*) embryonic days. (*a*′–*d*′) The representative PCNA immunostained transverse sections of the labyrinthine (*a*′,*b*′) or junctional (*c*′,*d*′) layers from PGDM placenta at E13.5 (*a*′,*c*′) or E18.5 (*b*′,*d*′) embryonic days. (*e*–*h*) The representative TUNEL immunostained transverse sections of the labyrinthine (*e*,*f*) or junctional (*g*,*h*) layers from control placenta at E13.5 (*e*,*g*) or E18.5 (*f*,*h*) embryonic days. (*e*′–*h*′) The representative TUNEL immunostained transverse sections of the labyrinthine (*e*′,*f*′) or junctional (*g*′,*h*′) layers from PGDM placenta at E13.5 (*e*′,*g*′) or E18.5 (*f*′,*h*′) PGDM embryonic days. (*i*,*j*) The bar charts compare the PCNA^+^ cell numbers of the labyrinth zone (*i*) or the junctional zone (*j*) from the control and PGDM placenta at E13.5 or E18.5 embryonic days. (*k*,*l*) The bar charts compare the TUNEL^+^ cell numbers of the labyrinth zone (*k*) or the junctional zone (*l*) of control and PGDM placenta at E13.5 or E18.5 embryonic days. (*m*) The western blotting data shows the protein expressions of P53 and PCNA from control and PGDM placenta at E13.5 or E18.5 embryonic days. (*n*,*o*) The bar charts show the ratios of the band arbitrary unit at the corresponding gene expression to the normalized β-actin from western blotting data in placental tissues at E13.5 (*n*) or E18.5 (*o*) embryonic days. Scale bars = 0.02 mm in (*a*–*h*,*a*′–*h*′).

PCNA was employed to assess whether placental cell proliferation was altered in the presence of high glucose levels (figure 3). The results were dissimilar at different developmental stages and different regions of the placenta. The PCNA^+^ cell numbers in the placental labyrinth zone were reduced at gestational age E13.5 (control: 192.70 ± 3.44, PGDM:173.70 ± 3.10, *p* < 0.01, *n* = 8 for each group; figure 3*a*,*b*,*i*) and increased at gestational age E18.5 (control: 182.20 ± 3.88, PGDM: 195.00 ± 4.12, *p* < 0.05, *n* = 8 for each group; figure 3*a*,*b*,*i*) in the presence of high glucose levels. Similarly, PCNA^+^ cell numbers in the placental junctional zone dropped at gestational age E13.5 (control: 209.90 ± 4.20, PGDM: 180.80 ± 3.77, *p* < 0.01, *n* = 8 for each group; figure 3*c*,*d*,*j*) but did not change significantly at gestational age E18.5 in the presence of high glucose levels (control: 244.70 ± 4.60, PGDM: 233.40 ± 5.21, *p* > 0.05, *n* = 7 for each group; figure 3*c*,*d*,*j*).

TUNEL staining was performed to examine cell apoptosis in the presence of high maternal levels of glucose (figure 3). The results demonstrated that TUNEL^+^ cells in the labyrinth zone were reduced at gestational ages E13.5 (control: 200.90 ± 6.12, PGDM: 184.10 ± 2.02, *p* < 0.05, *n* = 10) and E18.5 (control: 200.60 ± 2.85, PGDM: 184.10 ± 3.72, *p* < 0.01, *n* = 10), and TUNEL^+^ cells in the junctional zone were reduced at gestational age E18.5 (control: 227.80 ± 3.86, PGDM: 192.40 ± 6.60, *p* < 0.001, *n* = 10) in the presence of high glucose levels. However, TUNEL^+^ cells in the junctional zone at gestational age E13.5 (control: 196.10 ± 4.62, PGDM: 186.40 ± 3.71, *p* > 0.05, *n* = 10) did not change significantly in presence of high glucose levels. Moreover, we observed significant reduction of TUNEL^+^ cells in both the labyrinth and junctional zones at gestational age E18.5 compared with control (figure 3*e*,*f*,*k*–*l*). Meanwhile, western blotting was used to examine the expressions of P53 and PCNA at the protein level. The results of this showed that P53 expression was downregulated in the PDGM groups at both gestational ages E13.5 (*p* < 0.05, *n* = 3) and E18.5 (*p* < 0.01, *n* = 3), but PCNA expression was downregulated at gestational age E13.5 (*p* > 0.05, *n* = 3) and upregulated at gestational age E18.5 (*p* < 0.01, *n* = 3) compared with control (figure 3*m*–*o*). This implies that high levels of glucose interfere with normal placental cell proliferation and apoptosis.

### 3.3. Exposure to high levels of glucose promotes the differentiation of placental trophoblast cells in mice

**Figure 4.**
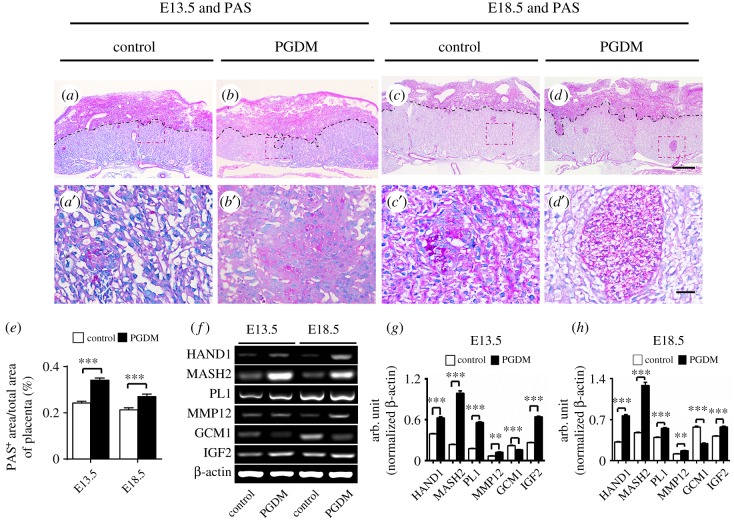
Glycogen-positive cell islets labelled by PAS increased in the junction zone and the labyrinth zone of PGDM placenta. (*a*–*d*) The representative PAS-stained transverse sections were taken from control (*a*,*c*) and PGDM (*b*,*d*) placenta at E13.5 (*a*,*b*) or E18.5 (*c*,*d*) embryonic days. (*a*′–*d*′) The high-magnification images were taken from the sites indicated by dotted squares in (*a*–*d*). (*e*) The bar chart compares the ratios of the PAS^+^ area to the total area in placental transverse sections taken from the control and PDGM groups at E13.5 and E18.5 embryonic days. (*f*) The RT-PCR data show the mRNA expressions of HAND1, MASH2, PL1, MMP12, GCM1 and IGF2 from control and PGDM placenta at E13.5/E18.5 embryonic days. (*g*,*h*) The bar charts show the ratios of the band arbitrary unit at the corresponding gene expression to the normalized β-Actin in placental tissues using RT-PCR data at E13.5 (*g*) or E18.5 (*h*) embryonic days. Scale bars = 0.5 mm in (*a*–*d*) and 0.05 mm in (*a*′–*d*′).

The PAS staining was performed to assess trophoblast cell differentiation in the placental labyrinth zone based on the observed positive PAS expression there. The results showed that the ratios of PAS^+^ area to total area in the labyrinth zone of PDGM placentas were significantly higher than in the control groups at both gestational ages E13.5 (control: 0.24 ± 0.01, PGDM: 0.34 ± 0.01, *p* < 0.001, *n* = 15) and E18.5 (control: 0.21 ± 0.01, PGDM: 0.27 ± 0.01, *p* < 0.001, *n* = 15; figure 4*a*–*e*). Another phenotype we observed in PDGM placenta in comparison to the control group is characterized by increased numbers of glycogen cell islets in the labyrinth and junctional zones, or by the protrusion of these cell islets from the labyrinth zone. This latter phenotype is shown in the high-magnification image below (figure 4*a*′–*d*′). Furthermore, we examined the mRNA expressions of trophoblast cell differentiation-related genes using RT-PCR in both the presence and absence of high glucose levels (figure 4*f*). The results demonstrated that the expressions of HAND1, MASH2, Placental lactogen-1 (PL1), MMP12 and IGF2 were upregulated in PDGM placenta at both gestational ages E13.5 (HAND1, *p* < 0.001; MASH2, *p* < 0.001; PL1, *p* < 0.001; MMP12, *p* < 0.01; GCM1, *p* < 0.001; IGF2, *p* < 0.001, *n* = 3 for each group) and E18.5 (HAND1, *p* < 0.001; MASH2, *p* < 0.001; PL1, *p* < 0.001; MMP12, *p* < 0.01; GCM1, *p* < 0.001; IGF2, *p* < 0.001, *n* = 3 for each group; figure 4*g*,*h*).

### 3.4. Exposure to high levels of glucose leads to the excess production of ROS in mouse placenta

**Figure 5.**
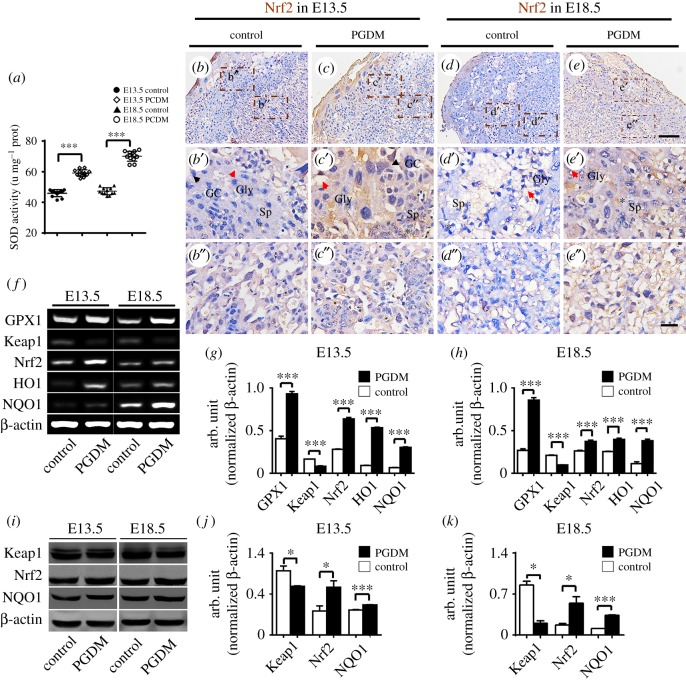
SOD and Nrf2 are upregulated in placental tissues in the presence of high levels of glucose. (*a*) The bar chart compares the SOD activities in placental tissues of the control (*b*,*d*) and PDGM (*c*,*e*) groups at E13.5 (*b*,*c*) and E18.5 (*d*,*e*) embryonic days. (*b*–*e*) The representative Nrf2 immunostained transverse sections came from control and PGDM placenta at E13.5/E18.5 embryonic days. (*b*′–*e*′) The high-magnification images were taken from the sites in the junction zone indicated by dotted squares in (*b*–*e*) (black arrow indicates the Gc, red arrow the Gly, asterisk the Sp). (*b*′–*e*′) The high-magnification images were taken from the sites in the labyrinth zone indicated by dotted squares in (*b*–*e*). (*f*) The RT-PCR data shows the mRNA expression of GPX1, Keap1, Nrf2, HO1 and NQO1 from control and PGDM placenta at E13.5/E18.5 embryonic days. (*g*,*h*) The bar charts show the ratios of the band arbitrary unit at the corresponding gene expression to the normalized β-actin in placental tissues from RT-PCR data at E13.5 (*g*) or E18.5 (*h*) embryonic days. (*i*) The western blotting data shows the expressions of Keap1, Nrf2 and NQO1 at high protein levels from control and PGDM placenta at E13.5/E18.5 embryonic days. (*j*,*k*) The bar charts show the ratios of the band arbitrary unit at the corresponding gene expression to the normalized β-actin in placental tissues from western blotting data at E13.5 (*j*) or E18.5 (*k*) embryonic days. Gc, trophoblast giant cells; Sp, spongiotrophoblast cell; Gly, glycogen cells. Scale bars = 0.15 mm in (*b*–*e*), 0.03 mm in (*b*′–*e*′) and 0.03 mm in (*b*″–*e*″).

In order to determine if oxidative stress is involved in the phenotypes described above, we performed a series of experiments to affect the production of ROS in the presence of high levels of glucose. First, we found that SOD (superoxide dismutase) activities (U mg^−1^ protein) in the PDGM groups occurred at much higher rate than in the control groups at both gestational ages E13.5 (control: 46.09 ± 0.98, PGDM: 58.94 ± 0.96, *p* < 0.001, *n* = 6) and E18.5 (control: 47.56 ± 1.02, PGDM: 70.17 ± 1.46, *p* < 0.001, *n* = 6; figure 5*a*). The immunostaining of Nrf2, a protein that plays an important role as an antioxidant, showed stronger expression in both the E13.5 and E18.5 PDGM groups than in the control groups (figure 5*b*–*e*). The diversity of Nrf2 expressions between the control and PDGM groups is distinct in the high-magnification images of the junctional (figure 5*b′*–*e*′) and labyrinth zones (figure 5*b*′–*e*′).

Second, we assessed the mRNA expressions of oxidative stress-related genes including GPX1, Keap1, Nrf2, HO1 and NQO1 in both the presence and absence of high glucose levels using RT-PCR (figure 5*f*). These results showed that all gene expressions went up in the PDGM group compared with the control group except for keap1. The expression of keap1, an inhibitor of Nrf2, was reduced among both the E13.5 (GPX1, *p* < 0.001; Keap1, *p* < 0.001; Nrf2, *p* < 0.001; HO1, *p* < 0.001; NQO1, *p* < 0.001, *n* = 3 for each group) and E18.5 (GPX, *p* < 0.001; Keap1, *p* < 0.001; Nrf2, *p* < 0.001; HO1, *p* < 0.001; NQO1, *p* < 0.001, *n* = 3 for each group) subjects. In order to confirm this observation, we assessed the protein expressions of several oxidative stress-related genes using western blotting (figure 5*i*). These results showed that the expressions of Keap1, Nrf2 and NQO1 in both E13.5 (Keap1, *p* < 0.05; Nrf2, *p* < 0.05; NQO1, *p* < 0.001, *n* = 3 for each group) and E18.5 (Keap1, *p* < 0.05; Nrf2, *p* < 0.05; NQO1, *p* < 0.001, *n* = 3 for each group) PDGM groups were also much higher than for the controls (figure 5*j*–*k*). All of these data suggest that oxidative stress is indeed activated in the environment of high glucose levels in mice.

### 3.5. Exposure to high glucose leads to excess autophagy in mouse placenta and BeWo cells

**Figure 6.**
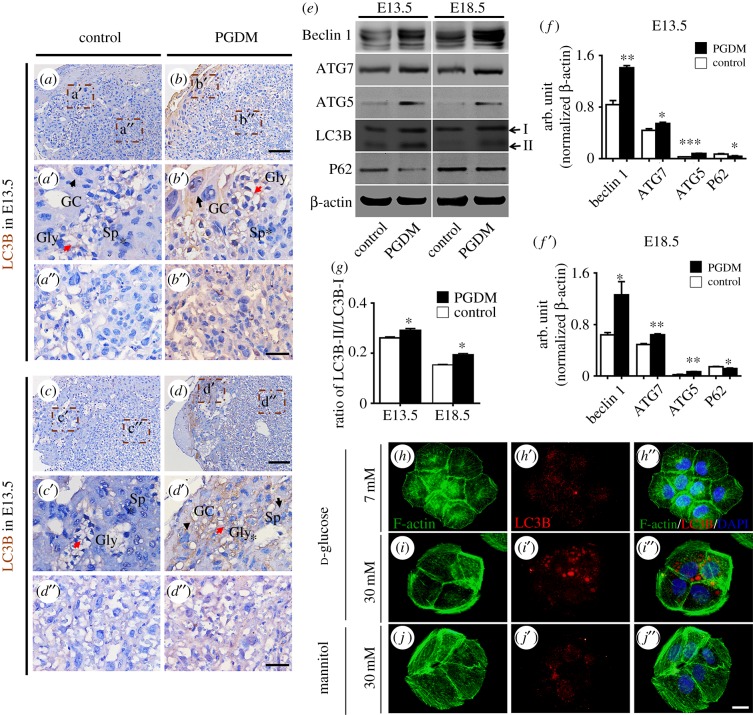
The detection of autophagy-related gene expressions in the presence of high levels of glucose. (*a*–*d*) The representative LC3B immunostained transverse sections from control (*a*,*c*) or PGDM (*b*,*d*) placenta at E13.5 (*a*,*b*)/E18.5 (*c*,*d*) embryonic days. (*a*′–*d*′) The high-magnification images were taken from the sites in the junction zone indicated by dotted squares in (*a*–*i*). (*a*′–*d*′) The high-magnification images were taken from the sites in the labyrinth zone indicated by dotted squares in (*a*–*d*). (*e*) The western blotting data show the expressions of Beclin1, ATG7, ATG5 and LC3B at high protein levels while the expressions of P62 at low protein levels from control and PGDM placenta at E13.5/E18.5. (*f*) The bar chart shows the ratios of the band arbitrary unit at Beclin1, ATG7, ATG5 and P62 expression to the normalized β-actin in placental tissues from western blotting data at E13.5 embryonic days. (*f*′) The bar chart shows the ratios of the band arbitrary unit at Beclin1, ATG7, ATG5 and P62 expression to the normalized β-Actin in placental tissues from western blotting data at E13.5 embryonic days. (*g*) The bar chart shows the ratios of LC3BII/LC3BI in placental tissues from western blotting data at E13.5 and E18.5 embryonic days. (*h*–*j*) The representative phalloidin stained F-actin from the control (*h*), the 30 mM d-glucose (*i*) or the 30 mM mannitol (*j*) treated BeWo cells, respectively. (*h*′–*j*′) The representative LC3B immunostained BeWo cells from the control (*h*′), the 30 mM d-glucose (*i*′) or the 30 mM mannitol (*j*′) group, respectively (black arrow indicates the Gc, red arrow indicates the Gly, asterisk indicates the Sp). (*h*″–*j*″) The amalgamated images of DAPI with *h*–*h*′, *i*–*i*′ and *j*–*j*′, respectively. Gc, trophoblast giant cells; Sp, spongiotrophoblast cell; Gly, glycogen cells. Scale bars = 0.15 mm in (*a*–*d*), 0.04 mm in (*a*′–*d*′) and 0.04 mm in (*a*″–*d*″).

In order to determine if autophagy is involved in the phenotypes described above, we performed a series of experiments to assess autophagy in presence of high glucose levels. The immunostaining of LC3B showed stronger expressions in both the E13.5 and E18.5 PDGM groups than in the controls (figure 6*a*–*d*). The diversity of LC3B expressions between the control and PDGM groups was distinct in the high-magnification images of junctional (figure 6*a*′–*d*′) and labyrinth zones (figure 6*a*′–*d*′). The western blotting results showed that the expressions of Beclin1, ATG7, ATG5 and the ratio of LC3B-II/LC3B-I in both the E13.5 (Beclin1, *p* < 0.01; ATG7, *p* < 0.05; ATG5, *p* < 0.001; LC3B-II/LC3B-I, *p* < 0.05, *n* = 3 for each group) and E18.5 (Beclin1, *p* < 0.05; ATG7, *p* < 0.01; ATG5, *p* < 0.01; LC3B-II/LC3B-I, *p* < 0.01, *n* = 3 for each group) PDGM groups were higher than in the controls, while the expressions of P62 in both the E13.5 (*p* < 0.05, *n* = 3 for each group) and E18.5 (*p* < 0.05, *n* = 3 for each group) PDGM groups were lower than in the controls (figure 6*e*–*g*). Meanwhile, LC3B immunofluorescent staining showed that the exposure to 30 mM d-glucose increased the rate of LC3B expression in BeWo cells compared with the 7 mM d-glucose control group (figure 6*h*–*j*″).

### 3.6. BeWo is employed to assess whether oxidation stress is involved in the differentiation of trophoblast cells

To further investigate the observations above, BeWo cells derived from human choriocarcinomas were employed to assess the nature of oxidative stress in the presence of high glucose levels. First, we examined the cell viability rate of BeWo cells in various concentrations of d-glucose and mannitol. This showed that high concentrations of d-glucose (17 and 30 mM) did not significantly change the cell viability of BeWo cells after incubation for 24 and 48 h periods in comparison to cells in the d-glucose control (7 mM) environment, but decreased the cell viability significantly at the 72 h mark (control and 17 mM: *p* < 0.01, control and 30 mM: *p* < 0.001, control and mannitol: *p* < 0.001, 30 mM and mannitol: *p* < 0.01, *n* = 6 for each group). At this point mannitol acts as an osmolarity control, and also has an inhibitive effect on cell viability of BeWo cells in the 30 mM d-glucose environment.

**Figure 7.**
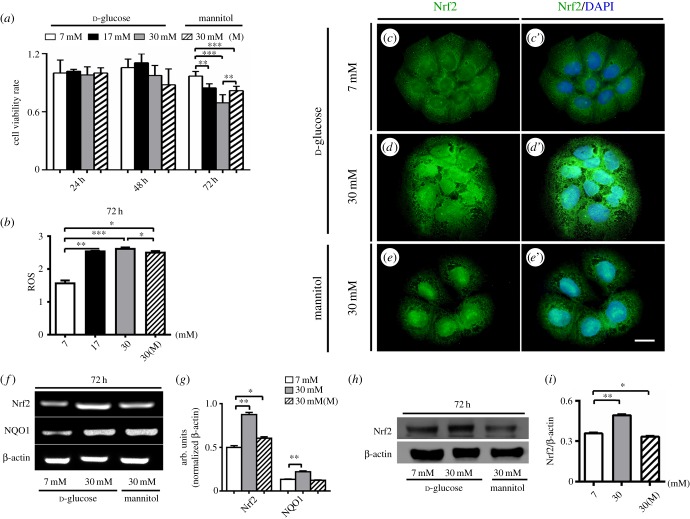
Nrf2 signalling of BeWo cell lines is activated in the presence of high levels of glucose. (*a*) The bar chart compares the BeWo cell viabilities (CCK8) in various concentrations of glucose (control—7 mM, 17 mM, 30 mM) and 30 mM mannitol (osmolarity control) at 24, 48 and 72 h incubation times. (*b*) The bar chart shows the intracellular ROS production after 72 h treatment with various concentrations of glucose and mannitol. (*c*–*e*) The representative Nrf2 immunofluorescent staining images of the *in vitro*-cultured BeWo cells in the presence of 7 mM glucose-(control) (*c*), 30 mM glucose (*d*) and 30 mM mannitol (*e*), respectively. (*c*′–*e*′) The amalgamated images of DAPI + (*c*–*e*), respectively. (*f*) The RT-PCR data show the mRNA expressions of Nrf2 and NQO1 from control or glucose/mannitol-exposed BeWo cells. (*g*) The bar chart shows the ratios of the band arbitrary unit at the corresponding gene expression to the normalized β-actin in control or glucose/mannitol-exposed BeWo cells from RT-PCR data. (*h*) The western blotting data show the expression of Nrf2 at the protein level from control or glucose/mannitol-exposed BeWo cells. (*i*) The bar chart shows the ratios of the band arbitrary unit at the Nrf2 expression to the normalized β-actin in control or glucose/mannitol-exposed BeWo cells from western blotting data. Scale bars = 0.03 mm in (*c*–*e*,*c*′–*e*′).

Second, we found that intracellular ROS production is dramatically enhanced in both high concentrations of d-glucose (17 and 30 mM) and a 30 mM concentration of mannitol in comparison with the control group (7 mM d-glucose) (control and 17 mM: *p* < 0.05, control and 30 mM: *p* < 0.001, control and mannitol: *p* < 0.05, 30 mM and mannitol: *p* < 0.05, *n* = 4 for each group) after a 72 h incubation period. Meanwhile, we also assessed the expression of Nrf2 anti-oxidant in a high glucose environment. Nrf2 immunofluorescent staining showed that exposure to both 30 mM d-glucose and mannitol environments increased Nrf2 expression in BeWo cells compared with the 7 mM d-glucose controls (figure 7*c*–*e*′). RT-PCR data similarly showed that 30 mM d-glucose and mannitol environments could enhance the expressions of Nrf2 and NQO1, a Nrf2-regulated enzyme, compared with the 7 mM d-glucose control group (Nrf2, control and 30 mM: *p* < 0.01; control and mannitol: *p* < 0.05; NQO1, control and 30 mM: *p* < 0.01, control and mannitol: *p* > 0.05, *n* = 3 for each group; figure 7*f*–*g*). The enhancement of Nrf2 expression in high glucose environments was confirmed using western blotting (Nrf2, control and 30 mM: *p* < 0.01, control and mannitol: *p* < 0.05, *n* = 3 for each group; figure 7*h*–*i*). These results imply that exposure to high levels of glucose could activate oxidative stress to a significant degree in BeWo cells.

### 3.7. Trophoblast cell differentiation and autophagy induced by high levels of glucose occur through the regulation of Nrf2 signalling

**Figure 8.**
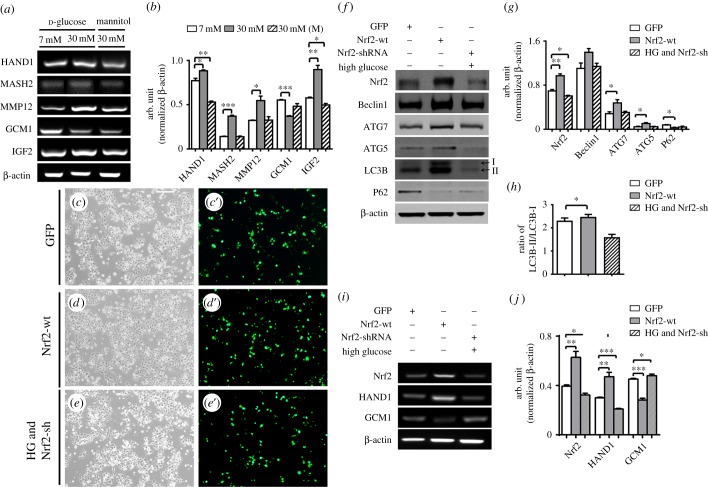
The expression and alteration of genes related to placental trophoblast differentiation and autophagy are induced by high levels of glucose through Nrf2 signalling in BeWo cell lines. (*a*) The RT-PCR data shows the mRNA expression of HAND1, MASH2, MMP12, GCM1 and IGF2 from control or glucose/mannitol-exposed BeWo cells. (*b*) The bar chart shows the ratios of the band arbitrary unit at the corresponding gene expression to the normalized β-actin in control or glucose/mannitol-exposed BeWo cells from RT-PCR data. (*c*–*e*) The representative bright-field images of the *in vitro*-cultured BeWo cells from GFP-transfected (*c*), Nrf2-wt-trasfected (*d*) and HG&Nrf2-shRAN-transfected (*e*). (*c*′–*e*′) The fluorescent images from (*c*–*e*), respectively. (*f*) The western blotting data shows the expression of Nrf2, Beclin1, ATG7, ATG5 and LC3B at high protein levels, while the expressions of P62 at low protein levels in GFP-, Nrf2-wt- or HG&Nrf2-shRAN-transfected control or glucose-exposed BeWo cells. (*g*) The bar chart shows the ratios of the band arbitrary unit at Nrf2, Beclin1, ATG7, ATG5 and P62 expression to the normalized β-actin in GFP-, Nrf2-wt- or HG&Nrf2-shRAN-transfected control or glucose-exposed BeWo cells from western blotting data. (*h*) The bar charts show the ratios of LC3BII/LC3BI in GFP-, Nrf2-wt- or HG&Nrf2-shRAN-transfected BeWo cells from western blotting data. (*i*) The RT-PCR data shows the mRNA expression of Nrf2, HAND1 and GCM1 in GFP-, Nrf2-wt- or Nrf2-shRAN-transfected control or glucose-exposed BeWo cells. (*j*) The bar chart shows the ratios of the band arbitrary unit at the corresponding gene expression to the normalized β-actin in GFP-, Nrf2-wt- or HG&Nrf2-shRAN-transfected BeWo cells from RT-PCR data. HG, high glucose. Scale bars = 0.2 mm in (*c*–*e*,*c*′–*e*′).

To investigate whether exposure to high levels of glucose affects trophoblast cell differentiation, we assessed the expression of differentiation-related genes in BeWo cells in the presence of high levels of glucose using RT-PCR. The results showed that high glucose levels (30 mM) increased the expressions of HAND1, MASH2, MMP12 and IGF2, and reduced GCM1 expression in comparison with the control group (HAND1, control and 30 mM: *p* < 0.05, control and mannitol: *p* < 0.01, *n* = 3 for each group; MASH2, control and 30 mM: *p* < 0.001, control and mannitol: *p* > 0.05; MMP12, control and 30 mM: *p* < 0.05, control and mannitol: *p* > 0.05; GCM1, control and 30 mM: *p* < 0.001, control and mannitol: *p* > 0.05; IGF2, control and 30 mM: *p* < 0.01, control and mannitol: *p* < 0.05, *n* = 3 for each group; figure 8*a*,*b*); in this context, mannitol acts as an osmolarity control. We then manipulated the Nrf2 gene expression levels in both the controls and the glucose-exposed BeWo cells through transfection with GFP (control), Nrf2-wt or HG&Nrf2-shRNA (figure 8*c*–*e*′); these results were confirmed by the western blotting technique (Nrf2, GFP and Nrf2-wt: *p* < 0.001, GFP and (HG&Nrf2-shRNA): *p* < 0.01, *n* = 3 for each group; figure 8*f*,*g*). The western blotting results showed the expression of Beclin1 (GFP and Nrf2-wt: *p* > 0.05, GFP and (HG&Nrf2-shRNA): *p* > 0.05, *n* = 3 for each group; figure 8*f*,*g*) did not change significantly in either the control group or the glucose-exposed BeWo cells through transfection with either Nrf2-wt or HG&Nrf2-shRNA. Meanwhile, the expressions of ATG7 and ATG5 in both the controls and the glucose-exposed BeWo cells through transfection with GFP (control), Nrf2-wt or Nrf2-shRNA, Nrf2-wt groups were higher than in the controls or HG&Nrf2-shRNA (ATG7, GFP and Nrf2-wt, *p* < 0.05, GFP and (HG&Nrf2-shRNA), *p* > 0.05; ATG5, GFP and Nrf2-wt, *p* < 0.05, GFP and (HG&Nrf2-shRNA), *p* > 0.05, *n* = 3 for each group; figure 8*f*,*g*). The expressions of P62 (GFP and Nrf2-wt, *p* < 0.05, GFP and (HG&Nrf2-shRNA), *p* > 0.05, *n* = 3 for each group) in Nrf2-wt groups were lower than in the controls or HG&Nrf2-shRNA (figure 8*f*,*g*). Meanwhile, the ratio of LC3B-II/LC3B-I in Nrf2-wt transfected BeWo cells at high protein levels was higher than the ratio observed in the controls (LC3B-II/LC3B-I, GFP& Nrf2-wt: *p* < 0.05, GFP and (HG&Nrf2-shRNA): *p* > 0.05, *n* = 3 for each group; figure 8*f*,*h*). In order to confirm the correlation between the oxidative stress-activated Nrf2 signalling and the alteration of trophoblast cell differentiation-related gene expressions, we manipulated the Nrf2 gene expression levels in the controls and the glucose-exposed BeWo cells through transfection with GFP, Nrf2-wt or Nrf2-shRNA; we then confirmed these results using RT-PCR (Nrf2, GFP and Nrf2-wt: *p* < 0.01, GFP and (HG&Nrf2-shRNA): *p* < 0.05; HAND1, GFP and Nrf2-wt: *p* < 0.01, GFP and (HG&Nrf2-shRNA): *p* < 0.001; GCM1, GFP and Nrf2-wt: *p* < 0.001, GFP and (HG&Nrf2-shRNA): *p* < 0.05, *n* = 3 for each group; figure 8*i*,*j*). These results showed that HAND1 was highly expressed when Nrf2 was over-expressed and minimally expressed when Nrf2 was knocked-down and exposed to high levels of glucose; that GCM1 expression was another way around as long as the Nrf2 gene manipulation (figure 8*i*–*j*).

## 4. Discussion

In the PGDM-induced mouse, we noted that fetal resorption occurred much more often than in control mice (figure 1*a*–*e*). Obviously, the predominant reason for fetal resorption is the presence of developmental defects in placenta; we confirmed this in mice with PGDM according to the previous reports [7]. Outwardly, placenta of mice with PGDM looks paler and weigh dramatically less than those in control mice, but there is not a significant difference in the placental diameters of the groups at gestational ages E13.5 and E18.5 (figure 1*f*–*k*). This implies that PGDM might affect the internal structures of placenta.

Therefore, we carefully compared the histological structures of transverse sections of placenta from both the control and PGDM groups. We found that the ratio of spongiotrophoblast cells to labyrinth cells at E13.5 and E18.5 in placenta of mice with PGDM was significantly increased compared with those same ratios in the placenta of controls (figure 2*a*–*e*). This suggests that PGDM caused the relative enhancement of the junctional zone and the relative reduction of the labyrinth zone in presence of high levels of glucose.

The labyrinth zone is known as the fetal portion of the placenta, while the junctional zone acts as the maternal portion of the placenta. Since the labyrinth is rich with vascular networks, we determined the expressions of angiogenesis-related genes in these zones. The results showed that VEGF and HIF1α expressions rose and VEGFR1 and FGFR2 expressions decreased in mice with PGDM (figure 2*f*–*h*), which may indicate that placental angiogenesis in the labyrinth zone is in a compensatory stage produced by impairment by high glucose levels.

Holzner *et al*. [23] studied the correlation between diabetes mellitus and histology of placenta in the past. After that, more evidence demonstrated that diabetes during pregnancy could cause structural and biochemical alterations in placental tissues, which in turn impact the normal physiological functions of placenta [24]. More recent literature even suggests that fetal hyperglycaemia leads to defects in placental angioarchitecture, which could initiate pathological responses such as added risk of cardiovascular diseases in fetal lateral life [25].

These pathological alterations in the placenta include dysfunctions of cell proliferation and apoptosis. Therefore, we used PCNA immunochemistry to determine the rate of cell proliferation. The results showed that cell proliferation decreased in both the labyrinth and junctional zones of the E13.5 PGDM placenta. By contrast, cell proliferation increased in the labyrinth zone and remained unchanged in the junctional zone in the E18.5 PGDM placenta (figure 3*a*–*d*,*i*,*j*). Meanwhile, TUNEL staining results showed that PGDM altered cell apoptosis in neither the labyrinth zone nor the junctional zone of the E13.5 placenta and reduced cell apoptosis in both zones in the E18.5 placenta (figure 3*e*–*h*,*k*,*l*). This tendency is generally confirmed by the western blotting results (figure 3*m*–*o*). The potential mechanisms of decreased apoptosis in the placentas from the PGDM placenta are as follows. First, p53-induced apoptosis requires an accumulation of ROS, so a strong anti-oxidant intracellular environment that could hinder the induction of apoptosis [26]. In our study, PGDM have excessive ROS, and anti-oxidant of Nrf2 is higher expression, so that might be one of the potential mechanisms. Second, autophagy is important in cell death decisions and can protect cells by preventing them from undergoing apoptosis [27]. However, the experimental results on cell proliferation and apoptosis in the presence of PGDM cannot explain the phenotypes that we observed above. This means that there must be other possible mechanisms involved.

Interestingly, we found more PAS^+^ staining protrusions extended from the junctional zone or from glycogen islets in the labyrinth zone in the PGDM placenta at both gestational age E13.5 and gestational age E18.5, implying that PGDM promotes trophoblast cell differentiation. In the PGDM mouse placenta, the expressions of many trophoblast differentiation-related genes including Hand1, Mash2, PL1, MMP12 and IGF2 increased; the exception was that expression of GCM1 which was reduced in comparison with the control group (figure 4*f*–*h*). Hand1 expression is limited to placental trophoblast cells and is requisite for differentiation and/or maintenance of trophoblast cells [28]. Mash2 is able to suppress the differentiation of trophoblast giant cells (TGC) [29]. PL1 and Placental lactogen- 2 (PL2) could act as the specific markers for TGC [30], and PLF works as a chemoattractant for endothelial cells in the maternal uterus [31].

For embryo implantation and trophoblast invasion, it is critical to maintain the close interaction between maternal decidual cells and fetal trophoblast cells. However, embryo implantation could not achieve this without the matrix metalloproteinases (MMPs) including MMP12 secreted by decidual cells [32]. The syncytiotrophoblast layer failed to fuse properly in Gcm1-deficient mouse placenta. Glial cells missing-1 (GCM1) plays the very important role in chorioallantoic development by identifying the folding sites of the chorionic plate and invagination of the allantoic mesoderm [33]. Placental IGF2 is deemed to modulate the development of mouse placenta as to diffusional exchange characteristics [34]. Thus, enhanced trophoblast cell differentiation in PDGM mice might be due to the alterations in gene expressions described above.

HIF1-α are involved in regulating the proliferation and differentiation of human trophoblast cells [31,35], implying that oxidative stress plays a role in placental development. Here, we found that the activity of SOD, one of the key antioxidant enzymes [36], increased in PDGM placenta compared with the controls (figure 5*a*). The Nrf2 is a transcription factor that acts as the crucial modulator of the redox homeostatic gene regulatory network. Immunostaining of Nrf2 showed that Nrf2 expressions in PDGM placentas at both gestational age E13.5 and gestational age E18.5 were stronger than those in the control placentas (figure 5*b*–*e*).

Meanwhile, the reduction of Keap1 expressions and increases in expressions of Nrf2, HO1 and NQO1 (the downstream genes of Nrf2) seen in PDGM placenta were shown using RT-PCR and western assays (figure 5*f*–*k*). These results indicate that oxidative stress is activated by diabetes mellitus. Furthermore, the imbalance of oxidative stress reactions was responsible for interfering with the expressions of trophoblast cell differentiation-related genes in the presence of high levels of glucose. This hypothesis could be partially confirmed by the reduction of the BeWo cell viability rate and by excess ROS production while Nrf2 signalling is activated in BeWo cells caused by high concentrations of glucose (figure 7).

Next, the correlation between the Nrf2 signalling and trophoblast cell differentiation could be revealed by the alteration of trophoblast cell differentiation-related gene expressions in BeWo cells in the presence of high levels of glucose (figure 8*a*–*b*). It is more easily conceivable that the expressions of HAND1 (which promotes chorion into ectoplacental cone or TGC) and GCM1 (which promotes chorion into labyrinth cells) changed as the expressions of Nrf2 genes in BeWo cells were manipulated (figure 8*c*–*g*).

Physiologically, autophagy is the process by which energy is supplied for embryonic development through the lysosomal degradation of cellular contents [37]. It has many functions in mammalian development including the development of placenta [38–40]. Under some pathological conditions including traumatic brain injury, ischaemia/reperfusion and tumour hypoxia, the relatively excessive accumulation of ROS could break cellular homeostasis, resulting in oxidative stress and mitochondrial dysfunction [41,42].

In this process, ROS could also promote the formation of autophagy [41]. Hung *et al*. [11] suggested that autophagy is important to protect trophoblast cells from injury caused by deficits in oxygen and glucose during pregnancy. Thus, we also assessed whether the autophagy was induced in the mouse placenta treated with high glucose and/or in the BeWo cells. Autophagy-related genes in the presence of high levels of glucose were detected in this study. People have found that autophagy-related genes (ATG) including ATG5, ATG7 and LC3 participate in the various stages of the autophagy process [43]. The importance of autophagy in embryo development has been confirmed by the mice with either Atg5 or Atg7 mutation could survive in the embryonic period but die soon after birth [44,45]. We found that the ratio of LC3B-II/LC3B-I was increased in groups treated with high levels of glucose compared with the control groups (figure 6*e*). We also discerned ATG5 and ATG7 are increased in PGDM groups compared with the control groups (figure 6*e*). When we transfected the Nrf2-wt, we discerned that the ratio of LC3B-II/LC3B-I was also increased compared with the GFP group. Meanwhile, the ratio of LC3B-II/LC3B-I in Nrf2-shRNA transfected BeWo cells at high protein levels was reduced, even after the BeWo cells were exposed to high levels of glucose (figure 8*f*,*h*). The same results appear in ATG5 and ATG7 (figure 8*f*,*g*). P62 decreased levels can be observed when autophagy is induced [46]. In our study, we have detected P62 was decreased in PGDM compared with the control (figure 6*e*,*f*′), and when we transfected the Nrf2-wt, we discerned that the ratio of P62 was also decreased compared with the GFP group. Meanwhile, the ratio of P62 in Nrf2-shRNA transfected BeWo cells at low protein levels was increased, even after the BeWo cells were exposed to high levels of glucose (figure 8*f*,*g*). So the decreased levels of P62 in our *in vivo* and *in vitro* experiments confirmed autophagy is increased in high-glucose environments. All these data suggest that Nrf2 signalling-activated autophagy might also be involved in the differentiation of trophoblast cells.

**Figure 9.**
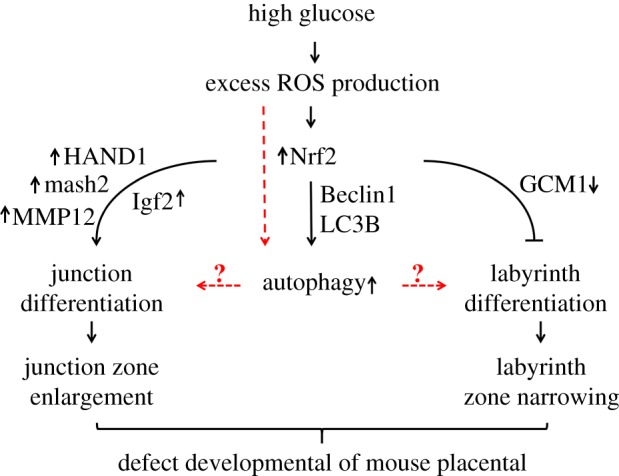
A proposed model that depicts the potential mechanisms for hyperglycaemia-induced defects in placenta.

In conclusion, we observed defects in placental development in mice with PGDM, which caused the relative reduction of the labyrinth zone and the enhancement of the junctional zone. Further experimental results imply that this might be correlated to the alteration of trophoblast cell differentiation-related gene expressions in high glucose settings rather than cell proliferation and apoptosis. Moreover, excess ROS production and dysfunction of Nrf2 signalling were confirmed as the causes of the alteration of trophoblast cell differentiation-related genes, as schematically shown in figure 9. In addition, ROS may also promote the formation of autophagy, and the imbalance of cell autophagy might also contribute to the observed placental phenotypes directly or indirectly, although the precise molecular biological mechanism by which it has this effect must still be explored in the future.

## Supplementary Material

Supplementary Fig S1

## Supplementary Material

Supplementary data-Data Availability

## References

[1] Ejdesjo A, Wentzel P, Eriksson UJ 2012 Influence of maternal metabolism and parental genetics on fetal maldevelopment in diabetic rat pregnancy. Am. J. Physiol. Endocrinol. Metab. 302, E1198–E1209. (doi:10.1152/ajpendo.00661.2011) 22374754 10.1152/ajpendo.00661.2011

[2] Gheorman L, Iliescu D, Ceausu I, Paulescu D, Plesea IE, Gheorman V 2011 Importance of early complex evaluation in high-risk pregnancy associated to diabetes mellitus. Case presentation and review of the literature. Rom. J. Morphol. Embryol. 52, 1127–1132. 22119836

[3] Macintosh MC, Fleming KM, Bailey JA, Doyle P, Modder J, Acolet D, Golightly S, Miller A 2006 Perinatal mortality and congenital anomalies in babies of women with type 1 or type 2 diabetes in England, Wales, and Northern Ireland: population based study. Br. Med. J. 333, 177 (doi:10.1136/bmj.38856.692986.AE) 16782722 10.1136/bmj.38856.692986.AE PMC1513435

[4] Corrigan N, Brazil DP, McAuliffe F 2009 Fetal cardiac effects of maternal hyperglycemia during pregnancy. Birth Defects Res. 85, 523–530. (doi:10.1002/bdra.20567) 10.1002/bdra.20567 19180650

[5] Zangen SW, Yaffe P, Shechtman S, Zangen DH, Ornoy A 2002 The role of reactive oxygen species in diabetes-induced anomalies in embryos of Cohen diabetic rats. Int. J. Exp. Diab. Res. 3, 247–255. (doi:10.1080/15604280214933) 10.1080/15604280214933 PMC2478588 12546278

[6] Huynh K, Kiriazis H, Du XJ, Love JE, Jandeleit-Dahm KA, Forbes JM, McMullen JR, Ritchie RH 2012 Coenzyme Q10 attenuates diastolic dysfunction, cardiomyocyte hypertrophy and cardiac fibrosis in the db/db mouse model of type 2 diabetes. Diabetologia 55, 1544–1553. (doi:10.1007/s00125-012-2495-3) 22374176 10.1007/s00125-012-2495-3

[7] Jarmuzek P, Wielgos M, Bomba-Opon D 2015 Placental pathologic changes in gestational diabetes mellitus. Neuro Endocrinol. Lett. 36, 101–105. 26071574

[8] Gauster M, Desoye G, Totsch M, Hiden U 2012 The placenta and gestational diabetes mellitus. Curr. Diab. Rep. 12, 16–23. (doi:10.1007/s11892-011-0244-5) 22102097 10.1007/s11892-011-0244-5

[9] Moruno F, Perez-Jimenez E, Knecht E 2012 Regulation of autophagy by glucose in mammalian cells. Cells 1, 372–395. (doi:10.3390/cells1030372) 24710481 10.3390/cells1030372 PMC3901114

[10] Adastra KL, Chi MM, Riley JK, Moley KH 2011 A differential autophagic response to hyperglycemia in the developing murine embryo. Reproduction 141, 607–615. (doi:10.1530/REP-10-0265) 21367963 10.1530/REP-10-0265 PMC3831622

[11] Hung TH, Hsieh TT, Chen SF, Li MJ, Yeh YL 2013 Autophagy in the human placenta throughout gestation. PLoS ONE 8, e83475 (doi:10.1371/journal.pone.0083475) 24349516 10.1371/journal.pone.0083475 PMC3862763

[12] Komatsu M, Ichimura Y 2010 Physiological significance of selective degradation of p62 by autophagy. FEBS Lett. 584, 1374–1378. (doi:10.1016/j.febslet.2010.02.017) 20153326 10.1016/j.febslet.2010.02.017

[13] Krishnan L, Nguyen T, McComb S 2013 From mice to women: the conundrum of immunity to infection during pregnancy. J. Reprod. Immunol. 97, 62–73. (doi:10.1016/j.jri.2012.10.015) 23432873 10.1016/j.jri.2012.10.015 PMC3748615

[14] Rossant J, Cross JC 2001 Placental development: lessons from mouse mutants. Nat. Rev. Genet. 2, 538–548. (doi:10.1038/35080570) 11433360 10.1038/35080570

[15] Watson ED, Cross JC 2005 Development of structures and transport functions in the mouse placenta. Physiology 20, 180–193. (doi:10.1152/physiol.00001.2005) 15888575 10.1152/physiol.00001.2005

[16] Cross JC 2000 Genetic insights into trophoblast differentiation and placental morphogenesis. Semin. Cell Dev. Biol. 11, 105–113. (doi:10.1006/scdb.2000.0156) 10873707 10.1006/scdb.2000.0156

[17] Kumar SD, Dheen ST, Tay SS 2007 Maternal diabetes induces congenital heart defects in mice by altering the expression of genes involved in cardiovascular development. Cardiovasc. Diabetol. 6, 34 (doi:10.1186/1475-2840-6-34) 17967198 10.1186/1475-2840-6-34 PMC2176054

[18] Han SS et al. 2015 Investigating the mechanism of hyperglycemia-induced fetal cardiac hypertrophy. PLoS ONE 10, e0139141 (doi:10.1371/journal.pone.0139141) 26418041 10.1371/journal.pone.0139141 PMC4587747

[19] Rampon C, Bouillot S, Climescu-Haulica A, Prandini MH, Cand F, Vandenbrouck Y, Huber P 2008 Protocadherin 12 deficiency alters morphogenesis and transcriptional profile of the placenta. Physiol. Genomics 34, 193–204. (doi:10.1152/physiolgenomics.00220.2007) 18477666 10.1152/physiolgenomics.00220.2007 PMC3305468

[20] Chen S, Sun FZ, Huang X, Wang X, Tang N, Zhu B, Li B 2015 Assisted reproduction causes placental maldevelopment and dysfunction linked to reduced fetal weight in mice. Sci. Rep. 5, 10596 (doi:10.1038/srep10596) 26085229 10.1038/srep10596 PMC4471727

[21] Acar N, Korgun ET, Cayli S, Sahin Z, Demir R, Ustunel I 2008 Is there a relationship between PCNA expression and diabetic placental development during pregnancy? Acta Histochem. 110, 408–417. (doi:10.1016/j.acthis.2007.11.011) 18377963 10.1016/j.acthis.2007.11.011

[22] De Simone R, Ajmone-Cat MA, Carnevale D, Minghetti L 2005 Activation of alpha7 nicotinic acetylcholine receptor by nicotine selectively up-regulates cyclooxygenase-2 and prostaglandin E2 in rat microglial cultures. J. Neuroinflammation 2, 4 (doi:10.1186/1742-2094-2-4) 15670336 10.1186/1742-2094-2-4 PMC548670

[23] Holzner JH, Thalhammer O 1965 On the histology and histochemistry of the placenta in diabetes mellitus and pregnancy glycosuria. Wien. Klin. Wochenschr. 77, 1024–1025. 5871992

[24] Diamant YZ 1991 The human placenta in diabetes mellitus. Isr. J. Med. Sci. 27, 493–497. 1960046

[25] Leach L 2011 Placental vascular dysfunction in diabetic pregnancies: intimations of fetal cardiovascular disease? Microcirculation 18, 263–269. (doi:10.1111/j.1549-8719.2011.00091.x) 21418381 10.1111/j.1549-8719.2011.00091.x

[26] Faraonio R, Vergara P, Di Marzo D, Pierantoni MG, Napolitano M, Russo T, Cimino F 2006 p53 suppresses the Nrf2-dependent transcription of antioxidant response genes. J. Biol. Chem. 281, 39 776–39 784. (doi:10.1074/jbc.M605707200) 10.1074/jbc.M605707200 17077087

[27] Thorburn A 2008 Apoptosis and autophagy: regulatory connections between two supposedly different processes. Apoptosis 13, 1–9. (doi:10.1007/s10495-007-0154-9) 17990121 10.1007/s10495-007-0154-9 PMC2601595

[28] Riley P, Anson-Cartwright L, Cross JC 1998 The Hand1 bHLH transcription factor is essential for placentation and cardiac morphogenesis. Nat. Genet. 18, 271–275. (doi:10.1038/ng0398-271) 9500551 10.1038/ng0398-271

[29] Tanaka M, Gertsenstein M, Rossant J, Nagy A 1997 Mash2 acts cell autonomously in mouse spongiotrophoblast development. Dev. Biol. 190, 55–65. (doi:10.1006/dbio.1997.8685) 9331331 10.1006/dbio.1997.8685

[30] Zaidi SK, Sullivan AJ, Medina R, Ito Y, van Wijnen AJ, Stein JL, Lian JB, Stein GS 2004 Tyrosine phosphorylation controls Runx2-mediated subnuclear targeting of YAP to repress transcription. EMBO J. 23, 790–799. (doi:10.1038/sj.emboj.7600073) 14765127 10.1038/sj.emboj.7600073 PMC380991

[31] Jackson LL, Colosi P, Talamantes F, Linzer DI 1986 Molecular cloning of mouse placental lactogen cDNA. Proc. Natl Acad. Sci. USA 83, 8496–8500. (doi:10.1073/pnas.83.22.8496) 3464966 10.1073/pnas.83.22.8496 PMC386957

[32] Benjamin MM, Khalil RA 2012 Matrix metalloproteinase inhibitors as investigative tools in the pathogenesis and management of vascular disease. Exp. Suppl. 103, 209–279. (doi:10.1007/978-3-0348-0364-9_7) 10.1007/978-3-0348-0364-9_7 PMC3367802 22642194

[33] Anson-Cartwright L, Dawson K, Holmyard D, Fisher SJ, Lazzarini RA, Cross JC 2000 The glial cells missing-1 protein is essential for branching morphogenesis in the chorioallantoic placenta. Nat. Genet. 25, 311–314. (doi:10.1038/77076) 10888880 10.1038/77076

[34] Sibley CP et al. 2004 Placental-specific insulin-like growth factor 2 (Igf2) regulates the diffusional exchange characteristics of the mouse placenta. Proc. Natl Acad. Sci. USA 101, 8204–8208. (doi:10.1073/pnas.0402508101) 15150410 10.1073/pnas.0402508101 PMC419581

[35] Mo FE, Muntean AG, Chen CC, Stolz DB, Watkins SC, Lau LF 2002 CYR61 (CCN1) is essential for placental development and vascular integrity. Mol. Cell. Biol. 22, 8709–8720. (doi:10.1128/MCB.22.24.8709-8720.2002) 12446788 10.1128/MCB.22.24.8709-8720.2002 PMC139880

[36] Li Y et al. 1995 Dilated cardiomyopathy and neonatal lethality in mutant mice lacking manganese superoxide dismutase. Nat. Genet. 11, 376–381. (doi:10.1038/ng1295-376) 7493016 10.1038/ng1295-376

[37] Aburto MR, Sanchez-Calderon H, Hurle JM, Varela-Nieto I, Magarinos M 2012 Early otic development depends on autophagy for apoptotic cell clearance and neural differentiation. Cell Death Dis. 3, e394 (doi:10.1038/cddis.2012.132) 23034329 10.1038/cddis.2012.132 PMC3481121

[38] Firulli AB, McFadden DG, Lin Q, Srivastava D, Olson EN 1998 Heart and extra-embryonic mesodermal defects in mouse embryos lacking the bHLH transcription factor Hand1. Nat. Genet. 18, 266–270. (doi:10.1038/ng0398-266) 9500550 10.1038/ng0398-266

[39] Giamas G et al. 2011 Kinome screening for regulators of the estrogen receptor identifies LMTK3 as a new therapeutic target in breast cancer. Nat. Med. 17, 715–719. (doi:10.1038/nm.2351) 21602804 10.1038/nm.2351

[40] Kraus JA, Dabbs DJ, Beriwal S, Bhargava R 2012 Semi-quantitative immunohistochemical assay versus oncotype DX((R)) qRT-PCR assay for estrogen and progesterone receptors: an independent quality assurance study. Mod. Pathol. 25, 869–876. (doi:10.1038/modpathol.2011.219) 22301704 10.1038/modpathol.2011.219

[41] Li L, Tan J, Miao Y, Lei P, Zhang Q 2015 ROS and autophagy: interactions and molecular regulatory mechanisms. Cell Mol. Neurobiol. 35, 615–621. (doi:10.1007/s10571-015-0166-x) 25722131 10.1007/s10571-015-0166-x PMC11486209

[42] Wang XJ, Li Y, Luo L, Wang H, Chi Z, Xin A, Li X, Wu J, Tang X 2014 Oxaliplatin activates the Keap1/Nrf2 antioxidant system conferring protection against the cytotoxicity of anticancer drugs. Free Radic. Biol. Med. 70, 68–77. (doi:10.1016/j.freeradbiomed.2014.02.010) 24556415 10.1016/j.freeradbiomed.2014.02.010

[43] Chen N, Karantza V 2011 Autophagy as a therapeutic target in cancer. Cancer Biol. Ther. 11, 157–168. (doi:10.4161/cbt.11.2.14622) 21228626 10.4161/cbt.11.2.14622 PMC3230307

[44] Komatsu M et al. 2005 Impairment of starvation-induced and constitutive autophagy in Atg7-deficient mice. J. Cell Biol. 169, 425–434. (doi:10.1083/jcb.200412022) 15866887 10.1083/jcb.200412022 PMC2171928

[45] Urbanek T, Kuczmik W, Basta-Kaim A, Gabryel B 2014 Rapamycin induces of protective autophagy in vascular endothelial cells exposed to oxygen-glucose deprivation. Brain Res. 1553, 1–11. (doi:10.1016/j.brainres.2014.01.017) 24462935 10.1016/j.brainres.2014.01.017

[46] Bjorkoy G, Lamark T, Pankiv S, Overvatn A, Brech A, Johansen T 2009 Monitoring autophagic degradation Of P62/Sqstm1. Method Enzymol. 452, 181–197. (doi:10.1016/S0076-6879(08)03612-4) 10.1016/S0076-6879(08)03612-4 19200883

