# Supplementary figures and images for: Nrf2 signalling and autophagy are involved in diabetes mellitus-induced defects in the development of mouse placenta
Source: Open Biol. 2016 Jul 6;6(7):160064. doi: 10.1098/rsob.160064 (PMC4967824; doi:10.1098/rsob.160064)

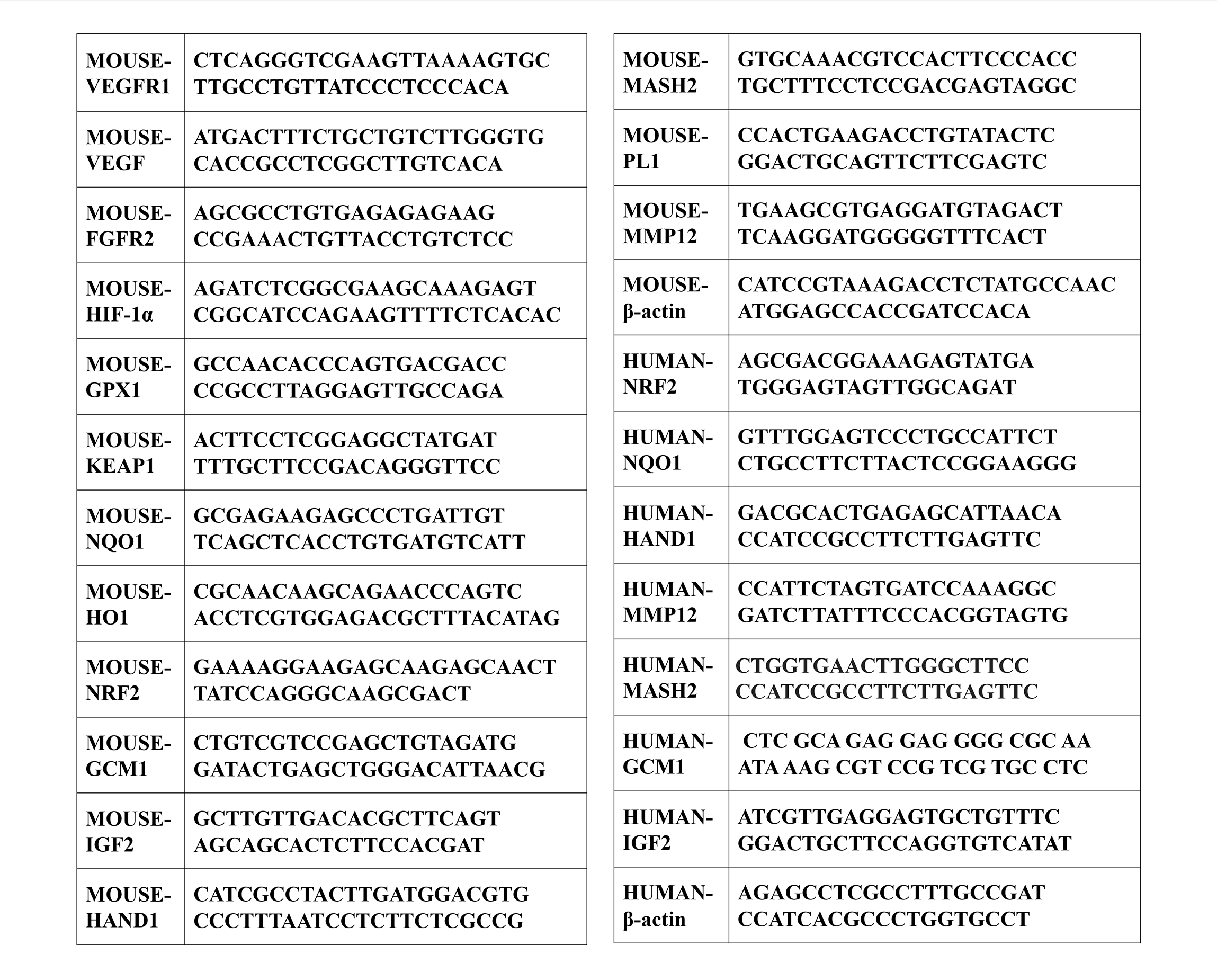

Supplement: Supplementary Fig S1 [file rsob160064supp1.tif]
