# Supplementary material for: Nrf2 signalling and autophagy are involved in diabetes mellitus-induced defects in the development of mouse placenta
Source: Open Biol. 2016 Jul 6;6(7):160064. doi: 10.1098/rsob.160064 (PMC4967824; doi:10.1098/rsob.160064)
Supplement: Supplementary data-Data Availability [file rsob160064supp2.docx]

**Data Availability**

**Figure 1**

**Fig. 1E** % of resorption

Control (N=34): 0.06 ± 0.02; PDGM (N=18): 0.15 ± 0.06, P<0.05.

**Fig. 1J** Placental weight

E13.5 (Control: 132.30 ± 2.34 mg, PGDM: 104.50 ± 2.08 mg, P<0.001. N=46 for each group);

E18.5 (Control: 194.20 ± 3.75mg, PGDM: 162.00 ± 6.20mg, P<0.001. N=21 for each group).

**Fig. 1K** Placental diameter(mm)

E13.5 (Control: 0.60 ± 0.15mm, PGDM: 10.17 ± 0.15mm, P>0.05. N=13 for each group);

E18.5 (Control: 12.10 ± 0.11mm, PGDM: 11.86 ± 0.07mm, P>0.05. N=13 for each group).

**Figure 2**

**Fig. 2E** Spong/Lab rations

E13.5 (Control: 0.74 ± 0.02, PGDM: 0.99 ± 0.03, P<0.001. N=25 for each group); E18.5 (Control: 0.52 ± 0.02, PGDM: 0.66 ± 0.04, P<0.01. N=25 for each group).

**Fig. 2G** Arbitrary (Normalized β-Actin)

E13.5 (VEGF- Control: 0.18 ± 0.002, PGDM: 0.41 ± 0.003, P<0.001; VEGFR1- Control: 0.42 ± 0.002, PGDM: 0.16 ± 0.003, P<0.001; FGFR2- Control: 0.67 ± 0.04, PGDM: 0.37 ± 0.01, P<0.01; HIF-1a- Control: 0.05 ± 0.002, PGDM: 0.79 ± 0.03, P<0.001. N=3 for each group);

**Fig. 2H** Arbitrary (Normalized β-Actin)

E18.5 (VEGF- Control: 0.13 ± 0.07, PGDM: 0.71 ± 0.04, P<0.001; VEGFR1- Control: 0.54 ± 0.01, PGDM: 0.30 ± 0.003, P<0.001; FGFR2- Control: 0.49 ± 0.06, PGDM: 0.16 ± 0.01, P<0.01; HIF-1a- Control: 0.09 ± 0.01, PGDM: 0.70 ± 0.02, P<0.001. N=3 for each group).

**Figure 3**

**Fig. 3I** PCNA Immunolabeling (PCNA in Labyrinth)

E13.5 (Control: 192.70 ± 3.44, PGDM: 173.70 ± 3.10, P<0.01. N=8 for each group);

E18.5 (Control: 182.20 ± 3.88, PGDM: 195.00 ± 4.12, P<0.05. N=8 for each group).

**Fig. 3J** PCNA Immunolabeling (PCNA in Junctional Zone)

E13.5 (Control: 209.90 ± 4.20, PGDM: 180.80 ± 3.77, P<0.01; N=8 for each group); E18.5 (Control: 244.70 ± 4.60, PGDM: 233.40 ± 5.21, P>0.05. N=7 for each group).

**Fig. 3K** TUNEL Immunolabeling (TUNEL in Labyrinth)

E13.5 (Control: 184.10 ± 2.02, PGDM: 200.90 ± 6.12, P<0.05. N=10);

E18.5(Control: 200.60 ± 2.85, PGDM: 184.10 ± 3.72, P<0.01. N=10)

**Fig. 3L** TUNEL Immunolabeling (TUNEL in Junctional Zone)

E13.5 (Control: 196.10 ± 4.612, PGDM: 186.4 ± 3.71, P>0.05. N=10);

E18.5 (Control: 227.80 ± 3.86, PGDM: 192.4 ± 6.60, P<0.001. N=10).

**Fig. 3N** Arbitrary (Normalized β-Actin)

P53: E13.5 (Control: 0.86 ± 0.14, PGDM: 0.29 ± 0.09, P<0.05. N=3)

PCNA: E13.5 (Control: 1.15 ± 0.10, PGDM: 1.04 ± 0.10, P>0.05. N=3)

**Fig. 3O** 3N Arbitrary (Normalized β-Actin)

P53: E18.5 (Control: 0.62 ± 0.10, PGDM: 0.12 ± 0.02, P<0.01. N=3).

PCNA: E18.5 (Control: 0.89 ± 0.03, PGDM: 1.19 ± 0.06, P<0.01. N=3)

**Figure 4**

**Fig. 4E** PAS area /Toltal area of placenta (%)

E13.5 (Control: 0.24 ± 0.01, PGDM: 0.34 ± 0.01, P<0.001. N=15)

E18.5 (Control: 0.21 ± 0.01, PGDM: 0.27 ± 0.01, P<0.001. N=15)

**Fig. 4G** Arbitrary (Normalized β-Actin)

E13.5 (HAND1- Control: 0.39 ± 0.01, PGDM: 0.63 ± 0.02, P<0.001; MASH2- Control: 0.24 ± 0.01, PGDM: 0.99 ± 0.04, P<0.001; PL1- Control: 0.18 ± 0.01, PGDM: 0.56 ± 0.01, P<0.001; MMP12- Control: 0.07 ± 0.003, PGDM: = 0.12 ± 0.01, P<0.01; GCM1- Control: 0.22 ± 0.01, PGDM: 0.16 ± 0.003, P<0.001; IGF2- Control: 0.27 ± 0.003, PGDM: 0.64 ± 0.01, P<0.001. N=3 for each group).

**Fig. 4H** Arbitrary (Normalized β-Actin)

E18.5 (HAND1- Control: 0.32 ± 0.01, PGDM: 0.77 ± 0.02, P<0.001; MASH2- Control: 0.48 ± 0.01, PGDM: 1.29 ± 0.05, P<0.001; PL1- Control: 0.40 ± 0.01, PGDM: 0.55 ± 0.01, P<0.001; MMP12- Control: 0.12 ± 0.01, PGDM: = 0.17 ± 0.01, P<0.01; GCM1- Control: 0.57 ± 0.02, PGDM: =0.29 ± 0.01, P<0.001; IGF2- Control: 0.42 ± 0.01, PGDM: 0.58 ± 0.01, P<0.001. N=3 for each group)

**Figure 5**

**Fig. 5A** SOD activity (U/mg prot)

E13.5 (Control: 46.09 ± 0.98, PGDM: 58.94 ± 1.00, P<0.001. N=6);

E18.5 (Control: 47.56 ± 1.02, PGDM: 70.17 ± 1.46, P<0.001. N=6).

**Fig. 5G** Arbitrary (Normalized β-Actin)

E13.5 (GPX1- Control: 0.41 ± 0.03, PGDM: 0.93 ± 0.03, P<0.001; Keap1- Control: 0.17 ± 0.002, PGDM: 0.08 ± 0.01, P<0.001; Nrf2- Control: 0.28 ± 0.004, PGDM: 0.64 ± 0.02, P<0.001; HO1- Control: 0.09 ± 0.002, PGDM: 0.53 ± 0.01, P<0.001; NQO1- Control: 0.07 ± 0.002, PGDM: 0.30 ± 0.01, P<0.001. N=3 for each group).

**Fig. 5H** Arbitrary (Normalized β-Actin)

E18.5 (GPX1- Control: 0.27 ± 0.02, PGDM: 0.86 ± 0.03, P<0.001; Keap1- Control: 0.21 ± 0.004, PGDM: 0.10 ± 0.001, P<0.001; Nrf2- Control: 0.28 ± 0.004, PGDM: 0.64 ± 0.02, P<0.001; HO1- Control: 0.26 ± 0.003, PGDM: 0.40 ± 0.01, P<0.001; NQO1- Control: 0.11 ± 0.02, PGDM: 0.38 ± 0.02, P<0.001. N=3 for each group)

**Fig. 5J** Arbitrary (Normalized β-Actin)

E13.5 (Keap1- Control: 1.10 ± 0.082, PGDM: 0.83 ± 0.008, P<0.05; Nrf2- Control: 0.41 ± 0.09, PGDM: 0.82 ± 0.11, P<0.05; NQO1- Control: 0.43 ± 0.01, PGDM: 0.57 ± 0.003, P<0.001. N=3 for each group).

**Fig. 5K** Arbitrary (Normalized β-Actin)

E18.5 (Keap1- Control: 0.98 ± 0.189, PGDM: 0.20 ± 0.042, P<0.05; Nrf2- Control: 0.17 ± 0.03, PGDM: 0.54 ± 0.11, P<0.05; NQO1- Control: 0.11 ± 0.002, PGDM: 0.34 ± 0.01, P<0.001. N=3 for each group)

**Figure 6**

**Fig.6F** Arbitrary (Normalized β-Actin)

E13.5 (Beclin1-Control: 0.84 ± 0.06, PGDM: 1.41 ± 0.03, P<0.01; ATG7-Control: 0.44 ± 0.03, PGDM: 0.54 ± 0.02, P<0.05; ATG5-Control: 0.03 ± 0.001, PGDM: 0.08 ± 0.002, P<0.001; P62-Control: 0.07 ± 0.01, PGDM: 0.04 ± 0.01, P<0.05. N=3 for each group).

**Fig.6F’** Arbitrary (Normalized β-Actin)

E18.5 (Beclin1-Control: 0.64 ± 0.04, PGDM: 1.26 ± 0.20, P<0.05; ATG7-Control: 0.49 ± 0.01, PGDM: 0.64 ± 0.01, P<0.01; ATG5-Control: 0.02 ± 0.01, PGDM: 0.07 ± 0.001, P<0.01; P62-Control: 0.15 ± 0.01, PGDM: 0.12 ± 0.01, P<0.05. N=3 for each group).

**Fig.6G** Ratio of LC3B-II/LC3B-I

E13.5 (Control: 0.26± 0.004, PGDM: 0.29 ± 0.007, P<0.05. N=3 for each group);

E18.5 (Control: 0.15 ± 0.002, PGDM: 0.19 ± 0.004, P<0.01. N=3 for each group).

**Figure 7**

**Fig.7A** cell viability rate

72-hour [Control (7 Mm D-Glucose): 0.97 ± 0.02, 17mM: 0.85 ± 0.02, 30 mM: 0.69 ± 0.03; 30 Mm Mannitol: 0.82 ± 0.02. Control & 17Mm: P<0.01, Control & 30 Mm: P<0.001, Control & Mannitol, P<0.001, 30 Mm & Mannitol, P<0.01. N = 6 for each group]

**Fig.7B** ROS

Control (7 mM D-Glucose): 1.56 ± 0.05, 17 mM: 2.54 ± 0.01, 30 mM: 2.62 ± 0.02; 30 mM Mannitol: 2.62 ± 0.02. Control & 17 Mm: P<0.05, Control & 30Mm: P<0.001, Control & Mannitol: P<0.05, 30 Mm & Mannitol: P <0.05. N= 4 for each group

**Fig.7G** Arbitrary (Normalized β-Actin)

Nrf2-Control (7 mM D-Glucose): 0.66 ± 0.10, 30 mM: 0.69 ± 0.12; 30 mM Mannitol:0.63 ± 0.12. Control & 30Mm: P<0.01; Control & Mannitol: P<0.05. N= 3 for each group;

NQO1- Control (7 mM D-Glucose): 0.15 ± 0.03, 30 mM: 0.16 ± 0.03; 30 mM Mannitol:0.17 ± 0.03. Control & 30Mm: P<0.01, Control & Mannitol: P>0.05. N=3 for each group]

**Fig.7I** Nrf2/β-Actin

Nrf2- Control (7 mM D-Glucose): 0.36 ± 0.01, 30 mM: 0.49 ± 0.01, 30 mM Mannitol: 0.33 ± 0.01. Control and 30Mm: P<0.01, Control and Mannitol: P<0.05. N=3 for each group

**Figure 8**

**Fig.8B** Arbitrary (Normalized β-Actin)

HAND1- Control (7 mM D-Glucose): 0.77 ± 0.026, 30 mM: 0.88 ± 0.010, 30 mM Mannitol: 0.53 ± 0.012. Control & 30 Mm: P<0.05, Control & Mannitol: P<0.01. N=3 for each group;

MASH2- Control (7 mM D-Glucose): 0.14 ± 0.004, 30 mM: 0.37 ± 0.010; 30 mM Mannitol: 0.14 ± 0.007. Control & 30 Mm: P<0.001, Control & Mannitol: P>0.05;

MMP12- Control (7 mM D-Glucose): 0.32 ± 0.004, 30 mM: 0.55 ± 0.051; 30 mM Mannitol: 0.33 ± 0.039. Control & 30 Mm: P <0.05, Control & Mannitol: P >0.05;

GCM1- Control (7 mM D-Glucose): 0.55 ± 0.006, 30 mM: 0.37 ± 0.008, 30 mM Mannitol: 0.48 ± 0.031. Control & 30 Mm: P <0.001, Control & Mannitol: P >0.05. Control & 30Mm: P <0.01, Control & Mannitol: P<0.05. N=3 for each group

IGF2-Control (7 mM D-Glucose): 0.58 ± 0.009, 30 mM: 0.9 ± 0.048, 30 mM Mannitol: 0.5 ± 0.018. Control & 30 Mm: P<0.01, Control & Mannitol: P<0.05;

**Fig.8G** Arbitrary (Normalized β-Actin)

Nrf2- GFP: 0.7±0.03, Nrf2-wt: 0.97 ± 0.04, Nrf2-shRNA: 0.61 ± 0.01. GFP & Nrf2-wt: P<0.01, GFP & Nrf2-shRNA: P<0.05;

Beclin1- GFP: 1.10 ± 0.10, Nrf2-wt: 1.40 ± 0.07, Nrf2-shRNA: 1.14 ± 0.06. GFP & Nrf2-wt: P>0.05, GFP & Nrf2-shRNA: P>0.05;

ATG7- GFP: 0.29 ± 0.03, Nrf2-wt: 0.48 ± 0.06, Nrf2-shRNA: 0.31 ± 0.02. GFP & Nrf2-wt: P<0.05, GFP & Nrf2-shRNA: P>0.05;

ATG5- GFP: 0.05 ± 0.01, Nrf2-wt: 0.11 ± 0.02, Nrf2-shRNA: 0.05 ± 0.01. GFP & Nrf2-wt: P<0.05, GFP & Nrf2-shRNA: P>0.05;

P62- GFP: 0.08 ± 0.01, Nrf2-wt: 0.03 ± 0.01, Nrf2-shRNA: 0.04 ± 0.01. GFP & Nrf2-wt: P<0.05, GFP & Nrf2-shRNA: P>0.05. N=3 for each group

**Fig.8H** Ratio of LC3B-II/LC3B-I

LC3B-II/LC3B-I- GFP: 2.02 ± 0.07, Nrf2-wt: 2.45 ± 0.13, Nrf2-shRNA: 1.57 ± 0.15. GFP& Nrf2-wt: P<0.05, GFP & Nrf2-shRNA: P>0.05. N=3 for each group

**Fig.8J** Arbitrary (Normalized β-Actin)

Nrf2- GFP: 0.4 ± 0.01, Nrf2-wt: 0.63 ± 0.05, Nrf2-shRNA: 0.32 ± 0.01. GFP & Nrf2-wt: P<0.01, GFP & Nrf2-shRNA: P<0.05; HAND1-GFP: 0.3 ± 0.004, Nrf2-wt: 0.47 ± 0.04, Nrf2-shRNA: 0.21 ± 0.01. GFP& Nrf2-wt: P<0.01, GFP & Nrf2-shRNA: P<0.001; GCM1- GFP: 0.45 ± 0.01, Nrf2-wt: 0.28 ± 0.02, Nrf2-shRNA: 0.48 ± 0.01. GFP & Nrf2-wt: P <0.001, GFP & Nrf2-shRNA: P <0.05. N=3 for each group.
